# Neurological disability in HTLV-1 infected individuals according to the initial clinical presentation: cohort study

**DOI:** 10.1186/1742-4690-8-S1-A62

**Published:** 2011-06-06

**Authors:** Davi Costa, Néviton Castro, Matheus Tannus, Michael Sundberg, André Muniz dos Santos, Silvany Braga, Anselmo Souza, Lorena Dantas, Katia Viana, Valéria Gusmão, Tânia Luna, Taís Dellavechia, Glória Orge, Edgar Carvalho

**Affiliations:** 1 Immunology, Universidade Federal da Bahia, Salvador, Bahia, Brazil

## Background

It’s estimated that 10 to 20 million people are infected with the HTLV-1 virus worldwide. Two principal diseases are associated with this virus: the Adult T-cell Lymphoma/Leukemia (ATLL), and the HTLV-1 Associated Myelopathy/ Tropical Spastic Paraparesis (HAM/TSP). The last one is a debilitating neurological disease characterized by a chronic progressive paraparesis, with autonomic and mild sensorial complains. There are also incomplete forms of HAM/TSP, sometimes called subclinical or oligosymptomatic. Those mild and moderate neurological presentations are found in almost one fourth of the HTLV-1 patients and they include neurogenic bladder syndromes, erectile dysfunction, isolated pyramidal signs and peripheral neuropathy.

## Objective

To describe the evolution of neurological disability in HTLV-1 infected individuals.

## Methods

Cohort study with 419 HTLV-1 infected individuals, divided in three groups according to the main clinical picture in to:

1. asymptomatic, defined as absence of any neurological symptoms or signs;

2. oligosymptomatic, defined by presence of neurogenic bladder, erectile dysfunction, minor pyramidal signs and neuropathy (isolated or in combination);

3. HAM/TSP, as defined by WHO criteria. Those patients had an annual follow up, including neurological examination. EDSS was utilized to measure neurological disability in various domains. Failure was considered as any worse on EDSS scale or death due to HTLV-1. Censored was considered as lost of follow up or death due other causes.

## Results

In 419 analyzed patients 67(18,76%) were diagnosed with HAM/TSP, 96(26,89%) as oligosymptomatics, 194(54,34%) as asymptomatics. There were 10 deaths on the cohort and the annual death rate was 0,59/100 patients. In survival analyses there were 71 failures with an incidence rate of 0,09 individuals/year. Kaplan-Meyer survival estimate curves with log-rank test showed a significant difference between the three curves (p<0.01), with incidence rates and time at risk of 0,2 and 125 in HAM/TSP 0,08 and 242 in neurological symptoms and 0,05 and 445 in asymptomatic. Cox regression showed that TNF-α higher than 541 pg/ml (ORs 1,95 IC 95% 1,14-3,36), female gender (ORs 2,38 IC 95% 1,34-4,22), and history of hypotiroidism (ORs 3,64 IC 95% 1,31-10,08) had a strong association with neurological decreased function.

## Conclusion

Neurological disability in HTLV-1 infected patient increase with time, and it is worse in groups with symptoms or HAM/TSP. Some factors proved to be associated with neurological disability and two of them (diabetes and hypotiroidism) is life treating and one (TNF-α) can be a focus of future pharmacotherapy interventions studies.

